# Inhibition and Calendar Age Explain Variance in Game Performance of Youth Soccer Athletes

**DOI:** 10.3390/ijerph19031138

**Published:** 2022-01-20

**Authors:** Florian Heilmann, Rainer Wollny, Franziska Lautenbach

**Affiliations:** 1 Movement Science Lab, Institute of Sport Science, Martin-Luther University Halle-Wittenberg, 06120 Halle (Saale), Germany; 2 Sport Psychology, Institute of Sport Science, Humboldt-Universität zu Berlin, 10115 Berlin, Germany; rainer.wollny@sport.uni-halle.de (R.W.); franziska.lautenbach@hu-berlin.de (F.L.); 3 Institute of Sport Psychology and Sport Pedagogy, Leipzig University, 04109 Leipzig, Germany

**Keywords:** executive functions, game performance, cognitive skill transfer, talent identification, soccer, cognition

## Abstract

The assessment of core executive functions (EFs; i.e., inhibition, working memory, cognitive flexibility) has often been presented as a diagnostic tool for evaluating cognitive functions in recent publications. For example, EFs are essential in soccer because players must quickly adapt, change strategies, and inhibit responses in rapidly changing game situations. Previous research has shown relations between (subjectively rated) game performance and the EFs of soccer players. Nevertheless, the previous studies’ samples were heterogeneous in their performance level (experts vs. amateurs), and the ratings were rather unsystematic (no validated rating protocol). Therefore, the current study aimed to predict soccer players’ game performance (i.e., systematically rated by coaches) with the help of EF performance. Therefore, we assessed the game performance (small-sided game, Game Performance Assessment Instrument [GPAI]) and EFs (inhibition: flanker task; working memory: 3-back task; cognitive flexibility: number-letter task) of 94 male soccer players (12–19 years old) from Germany’s highest competitive level. Multiple regression model results indicate that inhibition (i.e., flanker effect) and calendar age explain ~18% of players’ game performance variance. Results have to be interpreted with regard to the age-dependency of game performance and EFs. In conclusion, even though the results are based on a cross-sectional study, it appears that calendar age needs to be considered when assessing EFs.

## 1. Introduction

Due to executive functions (EFs; i.e., working memory, inhibition, cognitive flexibility), athletes are able to mentally play with ideas, take the time to consider what to do next, meet novel, unanticipated challenges, resist temptations, and stay focused [1]. EFs are considered an essential part of athletic performance [2]. Especially in soccer, the three EFs are relevant because players must quickly adapt, change strategies, and inhibit responses in rapidly changing game situations [3]. In the opposite sense, cognitive functions as a broad term, including EFs, have been shown to be determined by sport-specific movement expertise and level of performance [4,5], as well as by the characteristics of the conducted sports [2,5,6]. There is an ongoing debate about the mentioned impact of sport mode on EFs and the inverse EFs as a basis of sports performance. Thus, the aim of the current study was to investigate the connection between EFs and actual game performance in preadolescent and adolescent soccer players assessed with a systematic protocol (i.e., the modified Game Performance Assessment Instrument).

### 1.1. Executive Functions in Sport

Elite team sports players, including soccer players, need to have advanced skills in perceiving and processing a large amount of information, such as information regarding their surroundings and positioning and kinematic information about their opponents [7]. Situations in soccer and most other sports are never identical [8]. EFs regulate nonroutine situations and are relevant in goal-directed actions under novel response programming when dominant, habitual responses are apparent [9].

It is generally accepted that there are three core EFs. Inhibition (1) is the ability to disregard the distraction of competing information, stay focused, and resist making one response to instead make another [10,11]. Working memory (2) is described as the ability to hold information in one’s cache to be able to manipulate it and use it for further processing [12]. Finally, cognitive flexibility (3) is the ability to flexibly switch one’s perspectives, focus of attention, or response mappings [10].

EFs play a relevant role in sports. For instance, if a player wants to pass the ball to a teammate and an opponent covers the passing line, then the player must inhibit the first planned action (inhibition) and change the strategy (cognitive flexibility). Furthermore, it is advantageous for the player if, for example, he or she can memorize the positions of his or her teammates and opponents and make them available at short notice (working memory).

### 1.2. EFs and Age

EFs have been shown to be strongly dependent on calendar age [13,14]. In detail, EFs have been argued to progress through early and late childhood and adolescence [15]. Their improvement appears to coincide with growth spurts and the maturation of the frontal lobe structures. De Luca et al. [13] found relevant growth periods between birth and age two, from ages seven to nine, and a final spurt between 16 and 19 years of age. A similar pattern has been shown in youth soccer players (see [16,17]). The research group of Beavan et al. has postulated that the development of EFs is determined by the usual effects of the central nervous system’s growth and maturation and that sports experience is not the relevant contributor. However, when comparing athletes’ EFs between different types of sports, distinctions between athletes’ ability to inhibit can be detected (e.g., a higher inhibitory ability in soccer players than in gymnasts), which provides support for the idea that it is not only age that is relevant for the development of EFs (see [18]). Especially due to these divergent findings, both sports experiences and the type of sport, as well as calendar age, should be considered when examining EFs in soccer.

### 1.3. EFs and Sports Experience and Characteristics of Sports

Extensive research postulates the positive impact of physical activity on EFs [19,20,21], especially for age groups ranging from 6 to 13 years and over 50 years old [22]. Focusing on athletes, numerous authors have postulated that the development of EFs is dependent on the specific affordances and characteristics of the conducted sports [5,6,18,22,23,24]. For example, [23] showed that open-skill sport athletes outperform closed-skill sport athletes in inhibitory control. In a similar vein, the findings of Krenn et al. [18] also suggest that the expression of EFs might be favored more by the practice of strategic sports (e.g., soccer) than by static sports (e.g., gymnastics). The authors concluded that EFs might play a more prominent role in strategic sports and, thus, are being automatically more used and trained.

### 1.4. Transfer of Cognitive Skills

There is evidence that shows that elite athletes perform better than non-athletes regarding EF performance [25] in, for example, tennis [26], volleyball [27], basketball [28,29] and soccer [3,30,31]. These differences may be attributed to processes that are collectively referred to as cognitive skill transfer (CST).

The CST process is described as training in a cognitive task that could result in an improvement of a related untrained cognitive task. In other words, training in a specific environment could result in an improvement in the cognitive functions of the athlete [3,32]. CST models argue that every task affordance consists of different skills and/or specific proficiency.

Therefore, tasks with more skills and knowledge have the most substantial transfer effects [33]. This is an example of the so-called “narrow transfer” hypothesis. With respect to soccer, this means that the requirements of soccer (i.e., inhibition) lead to soccer players training with respect to inhibition more often. Thus, players might be better at, for example, performing inhibitory tasks on a computer (i.e., faster response times, fewer mistakes). The contrary hypothesis, i.e., the “broad transfer” hypothesis, postulates that practising a context-specific skill improves individual components of cognition and is present regardless of context [12]. However, the “narrow transfer” hypothesis postulates only that this cognitive skill will only be usable within the context in which it has been practised. Providing support for the “narrow transfer” hypothesis is early research on chess players who appear to have superior working memory regarding the arrangement of chess pieces. Nevertheless, their overall working memory is normal [34]. Furthermore, existing contradictory results show that video game training can, for example, improve simple reaction times in further tests [35]. These inconsistent findings give rise to debates about the “narrow” and “broad” transfer hypotheses.

### 1.5. Game Performance and Executive Functions

Most of the previous research focuses on the differences between novices and experts, as well as those between different sports, regarding EF performance ([36,37,38,39], Table A1). However, it is still unclear whether they predict or distinguish actual sport performance (e.g., measured or rated performance of soccer players). Therefore, understanding the relation between EFs and sports performance (e.g., points made in the following season; present study: the rating of game performance) is of high relevance, as only an established connection justifies the development and evaluation of specific interventions to potentially increase EFs.

To date, only a few studies have examined the relationship between EFs by systematically rating or measuring game or sports performance [36,37,38,39]. In detail, a study of Vestberg et al. [3] focusing on 20–29 year old soccer players (“high division” vs. “low division”) found a significant positive correlation (*r* = 0.540) between performance (i.e., goals and assistance during a game) and cognitive performance in the design fluency tests. The authors also detected a positive correlation between points made in the following season and coach-rated game intelligence (low to moderate; most evident for cognitive flexibility; [39]. In contrast, in ice hockey, no significantly correlated relationship was found between EFs, the rating of players’ game intelligence, and their ability to recognize the game pattern, make predictions and show flexibility in regard to decision-making by coaches (i.e., design-fluency test, trail-making test; [37]). Additionally, no relationship between on-ice performance (i.e., difference between own goals and opponent goals) and EF performance was found. Focusing on differences in EFs between players (M_age_ = 23.7), only the elite-level centre forwards scored significantly higher on EFs than did the players in other positions; no differences between elite players’ and lower-league players were found. In a similar vein, studies focusing on soccer players have shown no differences between high-level soccer players and the general population regarding domain-generic (EF-measures) and domain-specific EF performance [36]. On the other hand, young soccer players (aged 10–21) who were accepted into an elite youth program outperformed the rejected players in the Stroop test and the design-fluency test [38]. However, the EF measures could not predict the approval or rejection of the academy.

Overall, the findings of studies that have examined the relation of game performance (rated or justified by admission to a talent program) and EFs could be characterized as inconsistent [30,31]. One of the reasons for this outcome might be that none of the studies compared the EFs of athletes doing the same sport at different athletic performance levels (i.e., soccer players of a youth academy with varying game performances). The diagnostics in this field have the goal of differentiating between the players or athletes of one team and not only distinguishing between amateurs and professionals. The rating or description of the game performance of the previous approaches lacks systematics. There is no validated protocol used for the rating used in recent studies (i.e., [37]). A validated protocol in this context helps to underpin the relations between EFs and game performance. In addition, a differentiated game assessment could help to clarify the background of relations.

### 1.6. The Present Study

The current study aims to explore the predictive value of EFs of young, high-performing soccer players (i.e., elite youth academy members) and their age of game performance (Game Performance Assessment Instrument [GPAI]; Refs [40,41]). We hypothesize that EF performance (response time, accuracy) and age (based on Stuss et al. [14]) can explain the game performance of youth soccer players (1). However, we refrain from predictions with respect to the amount of variance that the factors might explain. Finally, and without clear hypotheses, we will explore EF relations to specific game performance parameters to provide as much information on a potential relationship as possible (2).

## 2. Materials and Methods

### 2.1. Participants

An a priori statistical power analysis (F-test, fixed effects, omnibus, one-way) was conducted for sample size estimation based on the effect size of Verburgh et al. (Ref [30]; *N* = 74, *d* = 0.35). Thus, with an alpha of 0.05 and a power of 0.95 for two groups, the sample size needed for the current study (calculated with G*Power 3.1) was *N* = 88.

Ninety-four youth soccer players were recruited from a youth academy (age: 12–18 years; U13: *n* = 12, U14: *n* = 9, U15: *n* = 18, U16: *n* = 17, U17: *n* = 8). The players took part in the study voluntarily. The study protocol was in accordance with the ethical standards of the APA and the Declaration of Helsinki. Informed consent was obtained from all individual participants included in the study. The characteristics of the players regarding age, body mass, and body height are shown in Table 1. The soccer players were drawn from the highest national leagues of their age groups (first division).

### 2.2. Measurement of Executive Functions

The three core executive functions were assessed using standardized computer tasks with the software Inquisit Lab 5 (Millisecond Software LLC, Seattle, WA, USA) on a 17-inch screen and a QWERTZ keyboard.

Inhibition was assessed using the flanker task [42], which has shown high reliability coefficients in previous research (Ref [43], incongruent trials [response time]: *r* = 0.879, congruent trials [response time]: *r* = 0.856). Participants were presented with five black arrows on a white background. The middle arrow was set as the target. The arrows either all pointed in the same direction (→→→→→, congruent trials), or the middle arrow pointed in the opposite direction (→→←→→, incongruent trials). The participants were asked to respond with the “E” button if the target arrow pointed to the right and the “I” button if it pointed to the left. The task consisted of four practice and 70 test trials which were divided into two blocks. The value for the construct of inhibition was the response time (ms) for correct responses and accuracy of responses for the congruent (same direction of arrows) and incongruent (opposite direction of arrows) target arrows. Additionally, the flanker effect was calculated by subtracting response time for congruent trials from the response time for incongruent trials [44]. Thus, a lower flanker effect, as well as shorter response times and higher accuracy, was an indication of superior inhibition. The task assesses the ability to inhibit incongruent information compared with, for example, the stop-signal task, where the participant has to inhibit a programmed movement response.

**Figure 1:**
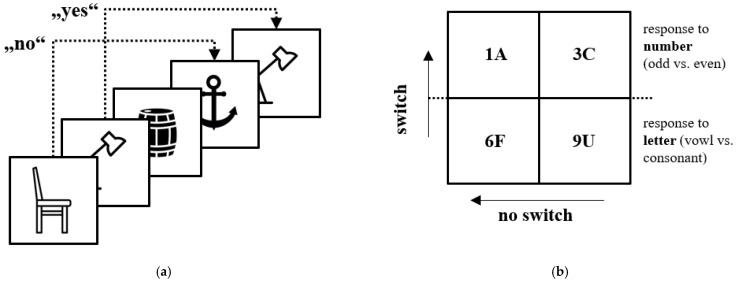
Stimulus presentation for the n-back task (**a**) and number-letter task (**b**).

A computerized three-back task (see Figure 1) was conducted to assess the working memory of participants based on Kirchner et al. [45], which has shown good reliability (Ref [46], response time for correct trials: *r* = 0.950). First, the test subjects were shown a series of pictures (emotionally neutral) that they had to recognize, and they had to decide if the actual symbol was presented three pictures ago. Then, the participants were given 23 test trials, followed by 46 target trials. More correct responses (accuracy) and faster response times to correct stimuli represent better working memory [44].

To assess cognitive flexibility, the number-letter task was modified from Rodgers et al. [47] by Miyake et al. [48]. Participants were presented with a 2 × 2 matrix in which a number and a letter (see Figure 1) appeared in one of the matrix fields. For the upper two fields, subjects had to respond to the letter (pressing the “E” key for a consonant vs. pressing the “I” key for a vowel). For the lower two boxes, they had to respond to the number by either pressing the “E” key for even numbers or the “I” key for odd numbers.

Participants were given 24 test trials for letters and numbers separately, followed by 24 number-letter test trials combined and 64 target trials, including 32 switch trials (i.e., switching from focusing on numbers to letters or vice versa) and 32 no-switch trials (i.e., continuing to focus on numbers or letters). Switch costs were calculated by subtracting the response time and accuracy of the responses of the switch trials from those of the no-switch trials. Lower switch costs, as well as shorter response times and high accuracy, were an indicator of superior cognitive flexibility. The task showed solid reliability in previous research (Ref [49], repetition trials [response time]: *r* = 0.773, switch cost [response time]: *r* = 0.676). Presented EF data was used in a previous study on the impact of maturation status, predicted adult body height and relative age effects on EFs in athletes [50].

### 2.3. Rating of Game Performance

Two professional soccer coaches (first league youth academy; holding a national coaching licence) rated the participants’ game performance using a modified version of the Game Performance Assessment Instrument (GPAI; Ref [40]). The coaches did not know the players beforehand. The GPAI was developed by [40] to examine game performance and thus describes the player’s ability to solve tactical problems and execute the appropriate skills. Therefore, the GPAI focuses on seven categories, such as “base”, “adjust”, “decisions made” or “skill execution” [40]. Coaches rate these categories on a 5-point Likert scale ranging from “very weak performance” (1 point) to “very effective performance” (5 points). The raters were trained in a 30 min session in using this specific rating scale. Raters were instructed to include the age-specific differences in their assessment. The interrater reliability for the total score was *r*_s_ (93) = 0.87 (*p* < 0.001; subscores: *r*_s_ (93) = 0.39, *p* < 0.001 to *r*_s_ (93) = 0.92, *p* < 0.001; see Table A2).

### 2.4. Procedure

The players were tested at their training facilities from September to November 2020. Players arrived at the facilities and were greeted by the experimenter (first author). They were instructed about the procedure and asked to sign the informed consent form. After that, they performed the cognitive tasks, which lasted approximately 45 min. The tasks were conducted in a randomized order.

At a second meeting, the players were rated in a small-sided game that had a duration time of 20 min each (5 vs. 5, no coach encouragement, field of 33 × 40.32 m). The modifications of the original test instrument related to the recording of the individual performances of the players. Because of the test economy, the modifications were not associated with each action, as in the publications of the developers of the instrument. Nevertheless, the modifications related to an overall impression of the coaches of the players’ performance in the various categories.

### 2.5. Statistical Analysis

Statistical analyses were carried out using SPSS 25 (SPSS, Chicago, IL, USA). Dependent and independent variables were checked for outliers. Outliers with an interquartile range over 3 were excluded from the analysis. Normality was checked for all dependent variables (Shapiro–Wilk test). Because of the nonnormality of various variables, we decided to exclude the variables that did not immediately indicate the underlying construct but rather led to the calculation of the main parameter (i.e., response time for congruent stimuli in the flanker task). The regression model that included nonnormally distributed variables did not lead to different results regarding the elucidation of variance.

We used a multiple linear regression model to investigate the predictive value of EFs (three-back task [response time, accuracy], flanker task [flanker effect], number-letter task [response time, accuracy]) and age of soccer players’ game performance. We ensured that the requirements for linear regression models were fulfilled. The relationship between the explanatory and dependent variables (game performance) was linear, and independent variables (EF measures and calendar age) did not have a linear relationship. Heteroscedasticity was checked using the Breusch–Pagan test. A partial correlation was calculated to show the direct connections between dependent (game performance) and independent variables (EF measures, calendar age). For all calculations, an initial level of significance was set at *p* < 0.05.

## 3. Results

### 3.1. Multiple Regression Model: Predicting Game Performance

The results of the regression indicated that the flanker effect and age (flanker task; inhibition) explained 18.1% of the variance in the rated game performance (*R*^2^ = 0.18, F(6,76) = 2.585, *p* = 0.025). The flanker effect (*β* = −0.005, *p* = 0.039) and calendar age (*β* = 0.080, *p* = 0.007) had significant effects in the multiple regression analysis.

### 3.2. Exploring Correlational Relations between EFs and Game Performance

**Figure 2:**
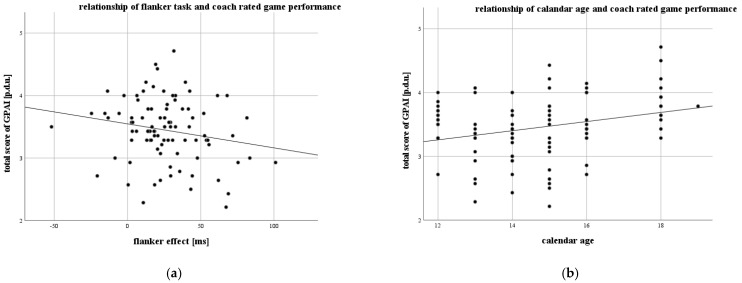
Partial relationship between flanker task performance (**a**) and calendar age (**b**) with coach rated game performance (GPAI).

There was a partial negative significant correlation between the flanker effect and coach-rated game performance, *r* = −0.224, *p* = 0.032; control variable: calendar age. Furthermore, there was a partial positive relationship between calendar age and coach-rated game performance, *r* = 0.308, *p* = 0.003; control variable: flanker effect. The correlations are displayed in Figure 2.

Further analysis showed partial correlations between the flanker effect and the subscores of the GPAI, as well as the calendar age and the subscores (Table A3). The flanker effect showed significant correlations with the subscores “support”, *r*_s_ = −0.240, *p* = 0.021) and “base” *r*_s_, = 0.199, *p* = 0.048. The subscores “skill execution”, *r*_s_ = 0.210, *p* = 0.042, “adjust” *r*_s_ = 0.240, *p* = 0.021, and “support” *r*_s_ = 0.240, *p* = 0.021 were positively correlated with calendar age.

## 4. Discussion

This study shows that calendar age and inhibition, as one of the core executive functions, explain 18.1% of the variance in coach-rated game performance (age: *r*_p_ = 0.308 *p* = 0.003, *R*^2^ = 0.112; flanker effect: *r*_p_ = −0.224, *p* = 0.032; *R*^2^ = 0.069) in youth soccer. Additionally, we found correlations between specific game performance parameters and EFs, as well as age. In our cross-sectional design, EFs were tested using n-back (working memory), flanker (inhibition), and number-letter tasks (cognitive flexibility).

### 4.1. Age and Inhibition Predict Game Performance

This study shows that calendar age and inhibition, as one of the core executive functions, explain 18.1% of the variance in coach-rated game performance (age: *r*_p_ = 0.308 *p* = 0.003, *R*^2^ = 0.112; flanker effect: *r*_p_ = −0.224, *p* = 0.032; *R*^2^ = 0.069) in youth soccer. So, our first hypothesis could be validated (1). Additionally, we found correlations between specific game performance parameters and EFs, as well as age. We did not predict any specific correlation between various items of the GPAI and EFs, but found significant correlations (2). In our cross-sectional design, EFs were tested using n-back (working memory), flanker (inhibition), and number-letter tasks (cognitive flexibility).

In accordance with our hypothesis (1), we found that age together with flanker performance predicted coach-rated soccer performance during a game of 5 vs. 5 players. In detail, within a sample of soccer players, calendar age and inhibition (flanker effect) predicted some variance of the players’ game performance rated by coaches. First, it needs to be clearly stated that the largest amount of variance was predicted by age. This is in line with previous research [36] and is not surprising, as our sample consisted of 12- to 19-year-olds; thus, the highly relevant growth periods between age 16 and 19 are part of our sample [13]. However, a small amount of variance in performance was also explained by EFs, particularly by inhibition.

To our knowledge, there has only been one study [38] so far that has tried to connect EFs to actual performance on the soccer field; thus, comparing our result to the majority of previous findings is somewhat difficult. However, our results are similar to previous findings that have shown a significant positive correlation between EFs (i.e., design-fluency test) and coach-rated game performance [36,39]. Furthermore, the findings of Beavan et al. [38] suggest a similar development of EFs (i.e., inhibition) in soccer players who are exposed to high-level soccer training compared to the general population, thereby providing evidence that age plays a relevant factor. However, we did not include a control group (e.g., general population) due to our aim of focusing on a homogeneous group of elite youth soccer players. Even though Beavan et al. [38] and Lundgren et al. [36] applied a different approach to relate the performance of ice hockey and soccer players with EFs, their results are similar to ours. In detail, they found positive correlations between the results from executive tests and the numbers of goals and assists made by players two seasons later [38]. In the same vein, a significant positive correlation was found between on-ice performance and the trail-making test (reflecting cognitive flexibility) scores [36].

That only inhibition and no other EFs were found to predict performance is somewhat surprising at first glance. However, considering theoretical approaches such as the unity-diversity framework of executive functions [48], this result seems comprehensible. The framework postulates that each EF is a combination of what is common to all three EFs (i.e., unity) and what is specific to each EF (i.e., diversity). Within this framework, there is no inhibitory specific factor; rather, inhibitory control is relevant and part of the cognitive flexibility and working memory. In other words, to be cognitively flexible, inhibitory control plays a crucial role, as only relevant information can be extracted from the environment, and irrelevant information must be inhibited. This basic ability, which is referred to as the regular EF, is necessary for all three EFs and is assessed using instruments measuring inhibition (see [48]). Therefore, our results might reflect only this common EF or the common ability to “actively maintain task goals and goal-related information” to predict performance (see [48], p. 11). On the other hand, this would mean that updating-specific factors (working memory) and shifting-specific factors (cognitive flexibility) might not be as relevant for predicting performance or that they might develop more strongly due to age-related growth (i.e., maturity) and should thus be included in the age factor (see [13]). This argument is, however, very speculative in nature. Future research on EFs in soccer and their relevance for performance needs a clear delimitation between the tasks that are used to measure EFs. To do so, clear theoretical directions and underpinnings need to be formulated.

Our exploratory analysis of correlations between EFs and performance indicates that there are two significant positive relationships between inhibition and game performance for “support” (i.e., off-the-ball movement to a position to receive a pass) and “base” (i.e., the appropriate return of the performer to a “home” or “recovery” position between skill attempts). These two tactical components of the overall game performance in a small-sided soccer game seem to be contributed to by the EFs, particularly by the players’ calendar age (2).

### 4.2. Study Limitations

Despite the large sample size, some limitations must be taken into account when interpreting the study. Even if the results of a previous study have shown that maturation and other development-related variables do not have to have an influence, certain influencing factors were not taken into account in the study design. For example, the IQ [51], physical fitness [52] or the effects of actual emotions [53] were not controlled for confounding. In detail, mental fatigue has been identified as an influencing factor on technical and decision-making performance in young soccer players during small-sided games (i.e., 5 vs. 5, Ref [54]). To consider this factor, sometimes referred to as resilient cognition (see [55]) should be controlled in future studies.

### 4.3. Practical Implications

The relationship between inhibition and calendar age with rated game performance (modified GPAI) suggests the practical implication, that the interpretation of EF measurements as performance diagnostics or talent identification always has to consider the calendar age of the players. However, the applied field could benefit from measuring assessing (motor) inhibition for diagnostics or talent identification because the calendar age and flanker effect can collectively explain 18.1% of the variance in coach-rated game performance.

## 5. Conclusions

As a conclusion regarding the multiple regression model results, calendar age must always be considered (see also [17]) when analysing EFs in athletes (1). Improvements in game performance and EFs could be determined by calendar age [38], as shown by the partial correlations in this study (2). The improvements in EFs could be explained by growth spurts and the maturation of the frontal lobe structures in preadolescence and adolescence [15]. The results have to be interpreted with caution because of the age-dependency of both the independent variable of EFs and the dependent variable of game performance.

## Figures and Tables

**Table 1:** Anthropometric data, characteristics, and EF-measure data of the soccer players (mean values and standard differences).

Anthropometric Data			M	SD
	age [years]		14.66	1.89
	Weight [kg]		58.74	12.72
	Height [cm]		170.52	12.03
Inhibition	flanker task (incongruent)	response time [ms]	466.24	69.27
	flanker task (congruent)	response time [ms]	442.48	72.66
	incongruent/congruent	accuracy [%]	97.55	0.03
	flanker effect	response time [ms]	26.63	25.27
Working Memory	n-back task	response time [ms]	825.22	317.82
		accuracy [%]	33.16	16.93
Cognitive Flexibility	number-letter task (switch)	response time [ms]	1454.50	302.88
		accuracy [%]	86.69	8.31
	number-letter task (no switch)	resonse time [ms]	1014.56	206.61
		accuracy [%]	95.70	3.20
	number-letter task (switch cost)	response time [ms]	437.66	197.68
		accuracy [%]	−7.56	7.30

## Data Availability

The data presented in this study are available on request from the corresponding author.

## Appendix A

**Table A1:** Studies examining the relations between executive functions (EFs) and game performance.

Authors and Year	Poulation	EF Measure	Measurement of Game Performance	Results
Vestberg et al. [39]	57 soccer players: male (*n* = 31) and female (*n* = 26) of “high division” (HD; M_age_ = 25.3; SD: 4.2) and “low division” (LD; M_age_= 22.8; SD: 4.1)	Design-Fluency test (DF), Stroop test (ST), Trail making test (TMT)	Numbers of goals and assists the players had scored two seasons later	1. Executive function test of athletes were sig. better than in norm group 2. HD players outperformed the LD players 3. Correlation test shows sig. correlation between the result from executive test and numbers of goals and assists of players scored two seasons later
Lundgren et al. [36]	50 ice hockey players: male, highest national level (*n* = 31, Level A), third-highest league (*n* = 19, Level B); M_age_ = 23.7; SD: 4.96	Design-Fluency test (DF), Trail making test (TMT)	Expert ratings game intelligence and a plus/minus statistic (goals of the team vs. goals against the team)	1. Elite players’ scores in EF tests were not sig. higher than those of lower-league hockey players 2. A significant correlation was found between on-ice performance and TMT scores 3. Elite-level center forwards scored sig. higher on DF than did players in other positions
Sakamoto et al. [37]	383 soccer players: male; M_age_ = 9.7	Design-Fluency test (DF), Stroop test (ST)	Admission to an elite youth program of a Japanese Football League club	1. Soccer players approved into the program got higher scores in general EFs tests than rejected players
Beavan et al. [38]	304 soccer players: male (age: 10–21 years)	Reactive stress tolerance task (RSTT); Stop signal task (SSRT); Precued choice response task (PCRTT)	Multiple object tracking task; Soccer-specific skills assessment task	1. Developmental trajectories of soccer players’ EFs follow the general populations’, despite long-term exposure to soccer-specific training and gameplay

**Table A2:** This is a table. Tables should be placed in the main text near to the first time they are cited.

Criteria		Interrater Reliability		Possible Ratings
		Spearman Rho Correlation (*r*_s_)	*p*-Value	
DMI	Decisions made: Making appropriate choices about what to do with the ball (or projectile) during the game	0.558	<0.001	5: Very effective performance 4: Effective performance 3: Moderately effective 2: Weak performance 1: Very weak performance
SEI	Skill execution: Efficient performance of selected skills	0.489	<0.001	
AI	Adjust: Movement of performer, either offensively or defensively, as required by the flow of the game	0.550	<0.001	
SI	Support: Off-the-ball movement to a position to receive a pass	0.927	<0.001	
GMI	Guard/mark: Defending an opponent who may or may not have the ball.	0.632	<0.001	
BI	Base: Appropriate return of performer to a “home” or “recovery” position between skill attempts	0.557	<0.001	
CI	Cover: Defensive support for the player making a play on-the-ball, or moving to the ball (or projectile)	0.389	<0.001	
Total score		0.870	<0.001	

**Table A3:** Spearman-rho partial correlations between flanker effect, calendar age, and the subscores of coach rated game performance (GPAI).

		Decisions Made (DMI)	Skill Execution (SEI)	Adjust (AI)	Support (SI)	Guard/Mark (GMI)	Base (BI)	Cover (CI)
Flanker effect	*r*_s_	−0.146	−0.044	−0.064	−0.240 *	−0.133	−0.199 *	0.190
	*p*-value	0.161	0.642	0.542	0.021	0.204	0.048	0.061
Calendar age	*r*_s_	0.189	0.210 *	0.303 **	0.240 *	0.145	0.188	0.022
	*p*-value	0.068	0.042	0.003	0.020	0.164	0.070	0.831

*: *p* < 0.05, **: *p* < 0.01.
